# Hydrous Cerium Dioxide-Based Materials as Solid-Contact Layers in Potassium-Selective Electrodes

**DOI:** 10.3390/membranes12040349

**Published:** 2022-03-22

**Authors:** Nikola Lenar, Robert Piech, Beata Paczosa-Bator

**Affiliations:** Faculty of Materials Science and Ceramics, AGH University of Science and Technology, Mickiewicza 30, PL-30059 Krakow, Poland; nlenar@agh.edu.pl (N.L.); rpiech@agh.edu.pl (R.P.)

**Keywords:** hydrous cerium dioxide, potassium determination, potentiometric sensing, hydrophobic materials, high electrical capacity, solid contact ion-selective electrodes

## Abstract

This paper introduces hydrous cerium dioxide applied for the first time as a solid-contact layer in ion-selective electrodes. Cerium dioxide belongs to the group of metal oxides that exhibit both redox activity and a large surface area and therefore was considered to be an appropriate material for the solid-contact layer in potentiometric sensors. The material was examined both standalone and as a component of composite materials (with the addition of carbon nanomaterial or conducting polymer). Three cerium dioxide-based materials were tested as solid-contact layers in potentiometric sensors in the context of their microstructure, wettability, and electrical properties. The addition of hydrous cerium dioxide was shown to enhance the properties of carbon nanotubes and poly(3-octylthiophene-2,5-diyl) by increasing the value of electrical capacitance (798 μF and 112 μF for hCeO_2_-NTs and hCeO_2_-POT material, respectively) and the value of contact angle (100° and 120° for hCeO_2_-NTs and hCeO_2_-POT material, respectively). The proposed sensor preparation method is easy, without the need to use an advanced apparatus or specific conditions, and fast; sensors can be prepared within an hour. Designed hCeO_2_-based electrodes exhibit competitive linear range and potential stability within the wide range of pH values (2.0–11.5). Designed electrodes are dedicated to potassium determination in environmental and clinical samples.

## 1. Introduction

The design and implementation of new functional materials for solid-contact layers in ion-selective electrodes is one of the most active topics in the scope of the potentiometry method [1]. In response to recent trends in potentiometric sensing, the new group of hydrous cerium dioxide-based materials was implemented into potassium-selective electrodes to improve their electrical and analytical parameters.

Among the electroactive materials for solid-contact layers in ion-selective electrodes we can distinguish high redox-capacity materials and high double-layer capacity materials [1,2].

In the first group we can find conducting polymers (represented by poly(3-octylthiophene-2,5-diyl) (POT) and poly(3,4-ethylenedioxythiophene) (PEDOT)) and molecular organic compounds (including organic molecular salts such as TCNQ and tetrathiafulvalene (TTF)).

In the second group, materials of large surface area can be found, including carbon nanomaterials (represented by carbon nanotubes) or nanostructure noble metals. According to this division, metal oxides can be classified into both groups, as they exhibit high redox activity and high surface area guaranteed by the nanometric size of the oxide particles [1].

Buck et al. [3] were the first to introduce metal oxides as sensing materials in pH-selective electrodes. As presented, PtO_2_, IrO_2_, RuO_2_, OsO_2_, Ta_2_O_5_, and TiO_2_ showed a near-Nernstian behavior in the pH range 2–12. The mechanism of sensing described and used by Buck was later taken advantage of by many groups, including the group of Paczosa-Bator when designing the metal oxide-contacted ion-selective electrodes. 

The first oxides to be implemented in the construction of ion-selective electrodes were ZnO [4] and CuO [4]. Later, Qin et al. introduced MoO_2_ microspheres [5], Yang et al. introduced MnO_2_ nanosheets [6], and our group, led by Paczosa-Bator, introduced ruthenium dioxide RuO_2_ and iridium dioxide IrO_2_ nanoparticles [7,8].

Metal oxides were shown to improve both the electrical and analytical properties of the sensors. Introducing ruthenium dioxide and iridium dioxide as solid-contact layers allowed the electrical capacity of the electrodes to increase and consequently improved the stability of the potentiometric response [7,8,9]. Both oxides were subsequently used as components of composite materials by mixing them together with carbon nanomaterials or conducting polymers. The addition of carbon nanomaterials ensures a greater electrical capacity of the electrodes in contrast to the standalone metal oxide applied as a solid-contact layer (which was presented in [10]). Conducting polymers were shown to improve the wetting properties of materials by elevating the contact angle value and making the solid-contact layer hydrophobic, according to [11].

In this paper we introduce a new metal oxide, namely hydrous cerium oxide as a solid-contact layer in potassium-selective electrodes. The oxide was used both as standalone material (hCeO_2_) and as a component of composite materials including hydrous cerium oxide-carbon nanotubes (hCeO_2_-NTs) and hydrous cerium oxide-conducting polymer (hCeO_2_-POT) materials.

The pH-sensing properties of cerium dioxide were described by Betelu et al. in [12] and the near-Nernstian response was achieved in a wide pH range (including highly alkaline solutions) due to its considerably high oxide-ion conductivity. These properties of oxide were taken advantage of in this work, and to the best of our knowledge this is the first paper to describe hydrous cerium dioxide as a solid-contact layer in ion-selective electrodes. 

What distinguishes potassium-selective sensors presented in the scope of this paper from other proposed solutions is the low cost and easy method of preparation.

## 2. Materials and Methods

### 2.1. Chemicals

The chemicals used for the purpose of this work include materials for the preparation of sensors and chemicals for the preparation of standard potassium solutions. 

Materials for sensor preparation include materials for solid-contact layers and ion-selective membrane. For solid-contact layers hydrous cerium dioxide (hCeO_2_), Multiwalled Carbon Nanotubes (NTs) and regiorandom poly(3-octylthiophene-2,5-diyl) (POT) purchased from Merck (previously Sigma-Aldrich, Saint Louis, MO, USA), Nanostructured and Amorphous Materials, Inc. (Houston, TX, USA) and Alfa Aesar (Haverhill, FL, USA), respectively, were used. The dimetylformamide (DMF) and tetrahydrofuran (THF) used as dispersants were obtained from POCH (Gliwice, Poland) and Merck, respectively. 

Membrane components included potassium ionophore I (Valinomycin), lipophilic salt—potassium tetrakis(4-chlorophenyl)borate (KTpClPB), 2-nitrophenyl octyl ether (o-NPOE), and poly(vinyl chloride) (PVC) were purchased from Merck (previously Sigma-Aldrich, Saint Louis, MO, USA) and dissolved in Tetrahydrofuran (Merck).

For the preparation of standard potassium solutions with K^+^ ions concentrations of 10^−^^7^ to 10^−^^1^ M, potassium chloride (KCl) purchased from POCH (Gliwice, Poland) was used. 

Hydrochloric acid and sodium hydroxide purchased from POCH (Gliwice, Poland) were used to adjust the pH value of the solutions during the pH sensitivity test.

### 2.2. Preparation of Sensors

Potentiometric sensors presented in the scope of this work are classified as solid-contact ion-selective electrodes and consist of two layers: a solid-contact layer placed directly onto the surface of glassy carbon disc electrodes and an ion-selective membrane. While a solid-contact layer should exhibit high electrical capacity and hydrophobicity, an ion-selective membrane is responsible for selective sensing of certain ions (in this case potassium).

For the purpose of this work, three materials based on hydrous cerium dioxide were prepared. The first material consisted of hydrous cerium dioxide (10 mg) dispersed ultrasonically in dimethylformamide (DMF) (1 mL). The other two materials were prepared with hydrous cerium dioxide and another compound, namely a carbon nanomaterial or conducting polymer, thereby creating composite materials. The first composite material was prepared by ultrasonically dispersing 5 mg of hCeO_2_ and 5 mg of multiwalled carbon nanotubes (NTs) in 1 mL of DMF. The second composite material consisting of metal oxide and conducting polymer was obtained by centrifugating 5 mg of hCeO_2_ and 5 mg of poly(3-octylthiophene-2,5-diyl) (POT) in 1 mL of tetrahydrofuran (THF). During the process, the POT dissolves in THF. After the centrifugation process (15 min, 5000 RPM) the residual THF was removed and the solid particles of metal oxide covered with polymer were dispersed ultrasonically in 1 mL of clean THF.

The materials preparation method is fast and easy with the use of basic laboratory equipment and does not require any special apparatus.

The potassium selective membrane was obtained by dissolving membrane components of total weight of 0.125 g: ionophore I 1.10% (*w*/*w*), KTpClPB 0.25% (*w*/*w*), oNPOE 65.65% (*w*/*w*) and PVC 33.00% (*w*/*w*) in 1 mL of THF.

In terms of sensors, glassy carbon disc (GCD) solid-contact potassium selective electrodes were obtained by casting the hCeO_2_-based materials onto the polished surface of the electrodes, evaporating the solvents (DMF and THF) at room temperature and subsequently covering the obtained solid-contact layers with potassium selective membrane solution (K^+^-ISM). The membranes are obtained by casting 60 μL of membrane solution and, after THF evaporation, the membrane layer is as thick as approximately 180 μm.

After drying, the prepared sensors were ready to use and conditioned in a 0.01 M KCl solution prior to every measurement. 

From the preparation of the electrode surface to the coating of them with an ion-selective membrane, the procedure is fast and easy, as both layers and membranes are applied with the use of the drop casting method. 

Together with three groups of solid-contact electrodes, one group of coated-disc electrodes (obtained by casting GCD electrodes with an ion-selective membrane) was prepared as a control group. The whole set of sensors included three items of sensors contacted with hCeO_2_ (GCD/hCeO_2_/K^+^-ISM), three items of sensors contacted with hCeO_2_-NTs (GCD/hCeO_2_-NTs/K^+^-ISM), three items of sensors contacted with hCeO_2_-POT (GCD/hCeO_2_-POT/K^+^-ISM) and three items of coated disc electrodes (GCD/K^+^-ISM).

### 2.3. Methods

Among the methods applied in the experiment for the examination of hCeO_2_-based materials contacted electrodes, the scanning electron microscope (SEM), contact angle microscope and chronopotentiometry method were used for the characterization of the materials and the potentiometry method was implemented to evaluate the influence of the presence of layers on the analytical performance of ion-selective electrodes. 

The microstructure of the hCeO2, hCeO_2_-NTs, and hCeO_2_-POT materials was investigated with a scanning electron microscope (model LEO 1530) from LEO Electron Microscopy, Carl Zeiss (Jena, Germany). The samples for this experiment were prepared by drop-casting alumina pads with material solutions. After drying, the pads were inserted into the microscope chamber. 

To examine the wetting properties of the obtained material, the contact angle microscope Theta Lite microscope with One Attention software by Biolin Scientific (Gothenburg, Sweden) was implemented into the studies. The wettability of materials was studied by dropping the water directly onto the surface of the electrode covered with a certain material. 

Both hCeO_2_-based materials and ready-to-use electrodes with ion-selective membrane were examined using the chronopotentiometric technique. For chronopotentiometric studies of materials, glassy carbon disc electrodes were covered with all materials examined (hCeO_2_, hCeO_2_-NTs, and hCeO_2_-POT) and placed in a measuring cell in sequence, together with the reference Ag/AgCl electrode with a 3 M KCl solution (type 6.0733.100 from ΩMetrohm, Herisau, Switzerland) and an auxiliary glassy carbon electrode. All electrodes were connected to the General Purpose Electrochemical System Autolab analyzer (AUT302N.FRA2-AUTOLAB) (ΩMetrohm Autolab, Utrecht, The Netherlands). A similar procedure was applied for the electrode examination. The cell was filled with 0.01 M KCl solution acting as an electrolyte. Chronopotentiograms (the potentiometric response of an electrode recorded with time under forced current conditions) were collected using NOVA 2.1 software. 

For the potentiometry method, all prepared ion-selective electrodes with hydrous cerium dioxide—based layers and potassium selective membrane and coated disc electrode were connected to the 16-channel potentiometer (Lawson Labs, Inc., Malvern, PA, USA) and measurements were performed against the Ag/AgCl electrode (type 6.0733.100 Ω Metrohm, Herisau, Switzerland) reference electrode in the presence of a platinum auxiliary electrode. 

## 3. Results

### 3.1. Materials Micostructure

The difference between the microstructure of hydrous cerium dioxide and two composite materials: hydrous cerium dioxide—carbon nanotubes and hydrous cerium dioxide—poly(3-octylthiophene-2,5-diyl) was examined using a scanning electron microscope.

**Figure 1:**
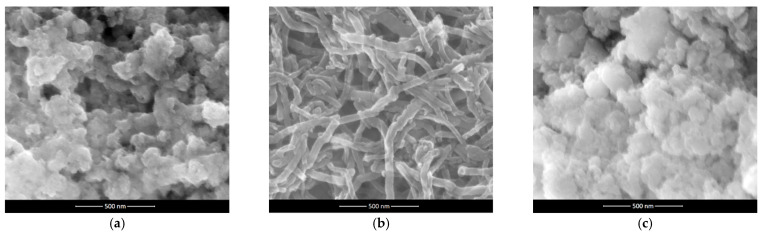
SEM scans of (**a**) hydrous cerium dioxide, (**b**) hydrous cerium dioxide—carbon nanotubes, (**c**) hydrous cerium dioxide—poly(3-octylthiophene-2,5-diyl). SEM experimental parameters—magnitude: 200,000×, detector: Helix, voltage: 18.0 kV, horizontal field width: 1.42 μm.

The scans obtained are presented in Figure 1 (a—hydrous cerium dioxide, b—cerium dioxide—carbon nanotubes, c—hydrous cerium dioxide—poly(3-octylthiophene-2,5-diyl)). Figure 1a shows the porous structure of the metal oxide with grains stacked in several layers. The size of single grain can be estimated at a few nanometers. Therefore, this material is characterized by a high surface area. 

Mixing two very different materials: metal oxide and carbon nanomaterial allowed to obtain the hCeO_2_-NTs composite material (Figure 1b) with oxide’s particles placed onto carbon nanotubes. This allowed for the enhancement of the porosity and surface area of the material in contrast to the standalone metal oxide. 

The second composite material (hCeO_2_-POT) is presented in Figure 1c. Similarly to the ruthenium dioxide-poly (3-octylthiophene-2,5-diyl) material [11], the polymer forms the amorphous “carrier” in which the oxide particles are located. The addition of a conducting polymer changes the structure of the material, what is presented in the scan (Figure 1c). In the presence of POT, cerium dioxide particles form greater agglomerates. However, its nanosized grains can still be visible and, as it is further proved during the chronopotentiometric test, it did not cause a reduction in their surface area. 

### 3.2. Materials Wettability

**Figure 2:**
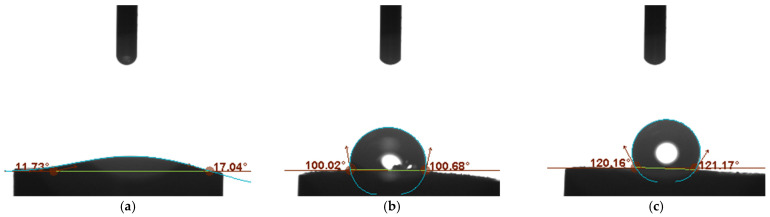
Contact angles of the studied materials: (**a**) hydrous cerium dioxide, (**b**) hydrous cerium dioxide—carbon nanotubes, (**c**) hydrous cerium dioxide—poly(3-octylthiophene-2,5-diyl).

The wetting properties of three studied materials based on hCeO_2_ were tested using a contact angle microscope. The water droplet of 4 μL was discharged onto the surface of the material and the contact angle between the tangent to the water drop and the surface of the material was calculated using the dedicated software. The results obtained are presented in Figure 2.

Unlike hydrophilic cerium dioxide (which is characterized by the contact angle of only 17°) (Figure 2a), the composite materials turned out to be superhydrophobic with the contact angle values of 100° and 120° for hCeO_2_-NTs (Figure 2b) and hCeO_2_-POT (Figure 2c) materials, respectively.

The hydrophobicity of the material is highly desired as it prevents the formation of a water layer on the surface of the solid-contact layer (under the membrane), ensuring the stability of the potentiometric response and the long lifetime of the electrode [13,14,15]. 

What should be emphasized here is that the presence of hydrophilic material (hCeO_2_) in the solid-contact layer does not cause the reduction of contact angle values of material; in contrast, the addition of cerium dioxide allowed us to obtain the superhydrophobic layer materials. The addition of hydrous cerium dioxide to the conducting polymer and carbon nanomaterial improved the hydrophobicity of both materials, as for standalone materials the achieved contact angle values of contact angles are: 89° for carbon nanotubes [10] and 90° for poly(3-octylthiophene-2,5-diyl) [11].

It may therefore be concluded that deigned hCeO_2_-based composite materials with their high contact angle values are perfect materials to be applied as solid-contact layers in ion-selective electrodes. 

### 3.3. Electrical Properties of Material

**Figure 3:**
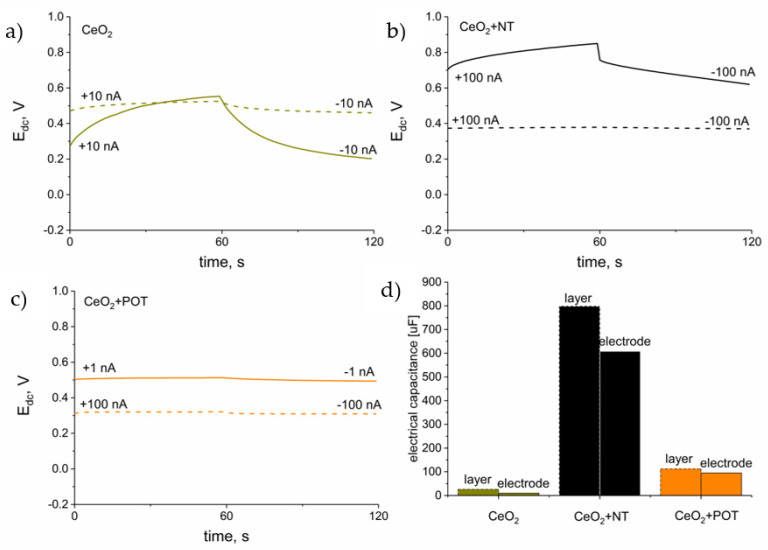
Chronopotentiograms of the studied materials and: (**a**) hydrous cerium dioxide (dark yellow line), (**b**) hydrous cerium dioxide—carbon nanotubes (black line), (**c**) hydrous cerium dioxide—poly(3-octylthiophene-2,5-diyl) (orange line). Solid-lined graphs refer to the solid-contact electrode and dash-lined ones—to solid-contact layers. Values of electrical capacitance parameter of electrodes and layers were compared on the diagram (**d**).

The electrical properties of hydrous cerium dioxide-based materials such as resistance and electrical capacity were examined using the chronopotentiometry method. This electrochemical technique allows one to calculate values of electrical parameters based on the potentiometric response to the set current. During the measurement, the current of 1, 10, or 100 nA (depending on the material properties) is forced to flow through the measuring cell and the potential response is recorded in six steps: for odd numbers of steps, the current has negative sing and for even numbers—positive sign. Electrical parameters are calculated based on the results obtained from each step using the R_total_ = △E_dc_/2I equation, where △E_dc_ stands for the value of the potential jumps between the odd and even steps and I is the set current value (for calculating the resistance R_total_ value) and C = I(dt/dE_dc_) equation, where E_dc_ stands for the potential value recorded over time (t) (for calculating the electrical capacitance parameter) [16]. Obtained chronopotentiograms (depicting first two steps) for each of the studied materials are presented in Figure 3 (dash lines). Electrical capacitance was calculated from all six steps for the linear parts of the recorded chronopotentiograms [17]. 

For the hydrous cerium dioxide layer, the value of electrical capacitance equal to 26.1 ± 0.6 μF. Much higher values were received for composite materials: 800 ± 10 μF and 113 ± 8 μF for hCeO_2_-NTs and hCeO_2_-POT materials, respectively. 

The resistance parameter is calculated on the basis of the value of potential jump between the two consecutive steps. The resistance values obtained for the studied materials were as follows: 267 ± 32 kΩ, 0.12 ± 0.07 kΩ and 1.32 ± 0.03 kΩ for hCeO_2_, hCeO_2_-NTs and hCeO_2_-POT materials, respectively. 

Therefore, it can be concluded that the hCeO_2_-NTs material is characterized by the most favorable electrical properties, as this material exhibits the highest electrical capacitance and the lowest resistance. 

### 3.4. Electrical Properties of Sensors

Ready-to-use electrodes with materials acting as solid-contact layers covered with ion-selective membrane were examined using the chronopotentiometry method according to the procedure described in the Section 3.3. 

Chronopotentiograms obtained after recording the potential response of electrodes under the set current (1, 10, or 100 nA) conditions are presented in Figure 3 (solid lines). Electrical parameters of electrodes: capacitance and resistance were calculated using mentioned in the previous section equations. Similarly, as for the solid-contact layers, the electrical capacitance parameter of the sensors was calculated for the linear parts of the chronopotentiograms.

The values of electrical parameters for each of the three groups of sensors examined are presented in Table 1.

The most favorable electrical properties can be attributed to the group of GCD/hCeO_2_ + NTs/K^+^-ISM electrodes for which, similarly as for the solid-contact layers, the highest electrical capacitance and one of the lowest resistances were received.

As can be seen in Figure 3d, the electrical capacitance parameter of the electrodes with ion selective membrane is in each case lower in contrast to the layers examined standalone without the polymeric membrane on the top.

The electrical capacitance value obtained for sensors with the hydrous cerium dioxide layer is not as high as for the other two oxides previously tested by the Paczosa-Bator group (0.93 and 1.07 mF for IrO_2_·2H_2_O [8] and RuO_2_·2H_2_O-contacted [18] electrodes). However, electrode composite materials with the addition of cerium oxide exhibit higher values in contrast to standalone carbon nanotubes (480 μF) [10] and poly(3-octylthiophene-2,5-diyl) (1.25 μF) [11].

What should be emphasized here is that hCeO_2_-based composite materials-contacted potassium-selective electrodes exhibit electrical properties competitive to the other materials presented so far in the literature with the preparation method being as fast and easy as possible.

### 3.5. Potentiometric Response

The potentiometric response towards potassium ions was investigated in standard potassium ions solutions with concentrations ranging from 10^−7^ to 10^−1^ M. Both the repeatability of the sensor response between three calibrations (*n* = 3 calibrations) performed on the same day (after 24 h of conditioning) and their reproducibility investigated within each group of electrodes (*n* = 3 items) were tested.

#### 3.5.1. Repeatability

**Figure 4:**
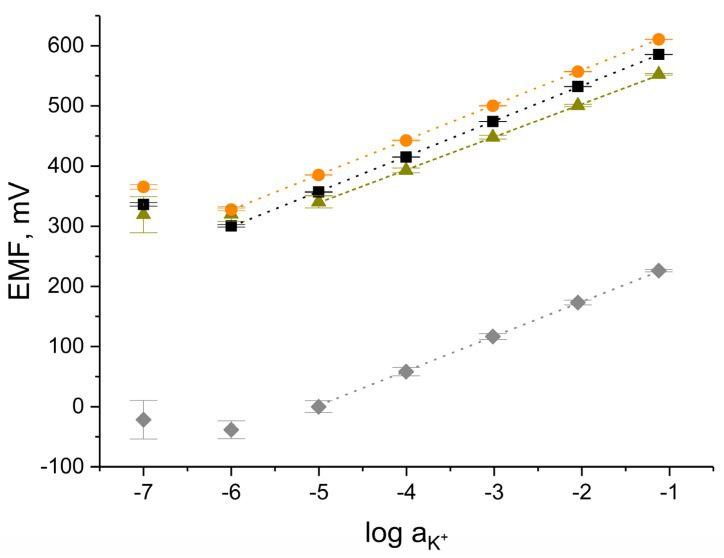
Calibration curves recorded for GCD/K^+^-ISM (⧫), GCD/hCeO_2_/K^+^-ISM (▲), GCD/hCeO_2_ + NTs/K^+^-ISM (■), GCD/hCeO_2_ + POT/K^+^-ISM (●) after 24 h of conditioning (*n* = 3 calibrations).

Figure 4 presents the average calibration curves (*n* = 3 calibrations) recorded for an electrode representing each group after 24 h of sensor conditioning in a 0.01 M KCl solution. 

Average values were calculated based on the results obtained during three calibrations performed one by one and standard deviation values were presented in the form of error bars. For sensors with composite hCeO_2_-NTs and hCeO_2_-POT solid-contact layers, standard deviation values equaled, respectively, to: 0.3 mV and 0.5 mV for the K^+^ ions concentrations between 10^−1^ and 10^−3^ M and not more 2 mV for lower concentrations (10^−5^, 10^−6^ M of K^+^ ions).

For the group of hCeO_2_-contacted electrodes and coated disc electrodes, the values are higher (accordingly up to 3 and 5 mV for higher concentrations and over 15 mV for lower concentrations for both groups).

Therefore, the best repeatability can be attributed to the GCD/hCeO_2_ + NTs/K^+^-ISM group of electrodes due to the shortest error bars.

#### 3.5.2. Reproducibility

The linear range of the potentiometric response of the sensors was indicated based on the slope value of the calibration curve (to reach the theoretical value given by the Nernstian equation). For the group of composite materials-contacted electrodes: GCD/hCeO_2_ + NTs/K^+^-ISM and GCD/hCeO_2_ + POT/K^+^-ISM, the linear range was set at 10^−6^ to 10^−1^ M of K^+^ ions and for the coated-disc and the group of GCD/hCeO_2_/K^+^-ISM electrodes the range was narrower: 10^−5^ to 10^−1^ M. The slope values of the calibration curves and the standard potential for each group of electrodes together with the standard deviation values (*n* = 3 items), which represent their reproducibility, are presented in Table 2.

For the lowest values of standard deviation, therefore, the greatest reproducibility can be attributed to the group of GCD/hCeO_2_ + POT/K^+^-ISM electrodes, which may indicate that within this group of electrodes the items are the most complementary. 

#### 3.5.3. Stability of Potentiometric Response 

**Figure 5:**
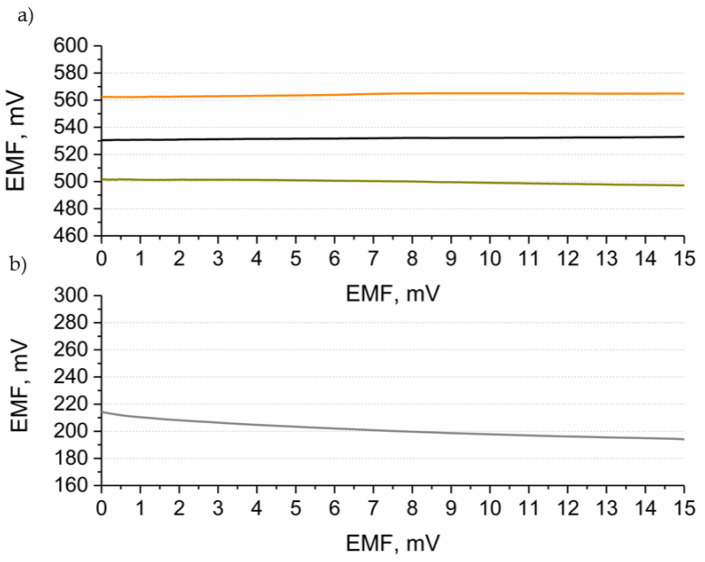
Potentiometric response recorded for (**a**): GCD/hCeO_2_/K^+^-ISM (dark yellow line), GCD/hCeO_2_ + NTs/K^+^-ISM (black line) and GCD/hCeO_2_ + POT/K^+^-ISM (orange line) electrode and (**b**): GCD/K^+^-ISM (grey line) electrode during 15 h-long measurement in 0.01 M KCl solution.

One of the most crucial parameters of sensors is the stability of their potentiometric response. The stability of the electrodes was tested during typical long-term potentiometric measurement (15 h) in 0.01 M KCl solution in a reference to a single junction potential electrode (Ag/AgCl electrode, ΩMetrohm, Switzerland). The stability of electrodes representing each group was compared on the basis of the values of the potential drift calculated as a change in potential—time (15 h) ratio (Figure 5).

The potential drift values were as follows: 0.086 mV/h, 0.095 mV/h, 0.24 mV/h and 1.22 mV/h for the GCD/hCeO_2_/K^+^-ISM, GCD/hCeO_2_ + NTs/K^+^-ISM, GCD/hCeO_2_ + POT/K^+^-ISM and GCD/K^+^-ISM electrode, respectively.

Low values of potential drift of hCeO_2_-based composite materials are ensured by high values of contact angle and high electrical capacity of layers. For hCeO_2_-POT-contacted electrode the stability of potentiometric response is almost as good as for the hCeO_2_-NT-contacted electrode of higher electrical capacity, thanks to the great hydrophobicity of the layer.

The lowest potential drift value characterizes the GCD/hCeO_2_ + NTs/K^+^-ISM group of electrodes, what is guaranteed by the high electrical capacity attributed to this group. As proved here, the higher the electrical capacitance parameter, the more stable the potentiometric response as this property of electrodes decides of their insensitivity to the perturbations that may occur during the measurement (such as power fluctuations or the current flow). 

#### 3.5.4. Light Sensitivity Test 

**Figure 6:**
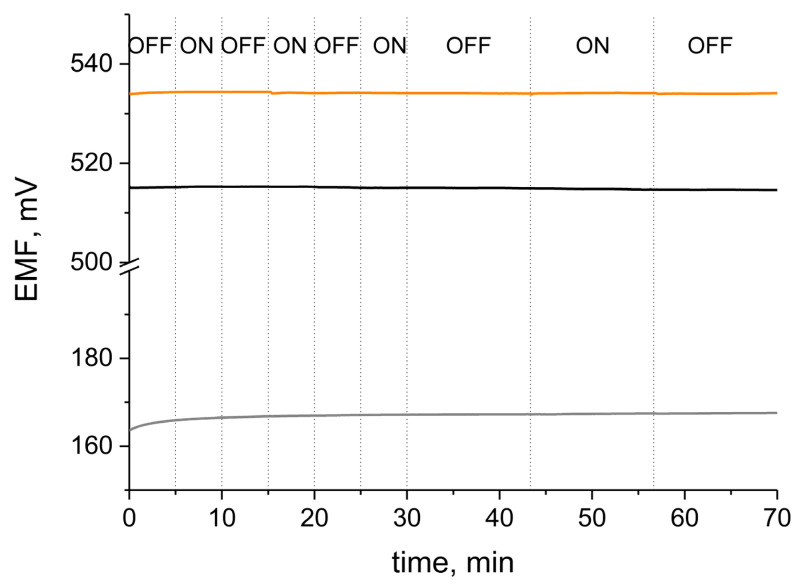
Potentiometric response recorded for GCD/K^+^-ISM (grey line), GCD/hCeO_2_ + NTs/K^+^-ISM (black line) and GCD/hCeO_2_ + POT/K^+^-ISM (orange line) electrode during the light test (with light ON and OFF).

As conducting polymers tend to be susceptible to light exposure [1], the light sensitivity test was performed for designed solid contact electrodes with POT as a component of the composite material (GCD/hCeO_2_ + POT/K^+^-ISM) in the presence of the GCD/K^+^-ISM and GCD/hCeO_2_ + NTs/K^+^-ISM electrodes used in this experiment as control groups. The test was performed in the standard solution of 0.01 M K^+^ ions and the potentiometric response of the electrodes was monitored while changing the light intensity: from very bright room light to complete dark conditions, followed again by light conditions. For the first 30 min of measurement, changes were more frequent and in the second part the exposure time was longer, as can be seen in Figure 6.

As presented in Figure 6, the potential response of the hCeO_2_-POT-contacted electrode was stable during the experiment and there was no reaction to the light exposure (not for this group or for other tested groups). The test confirmed that the electrodes presented in this paper are insensitive to light exposition.

#### 3.5.5. pH Sensitivity Test

**Figure 7:**
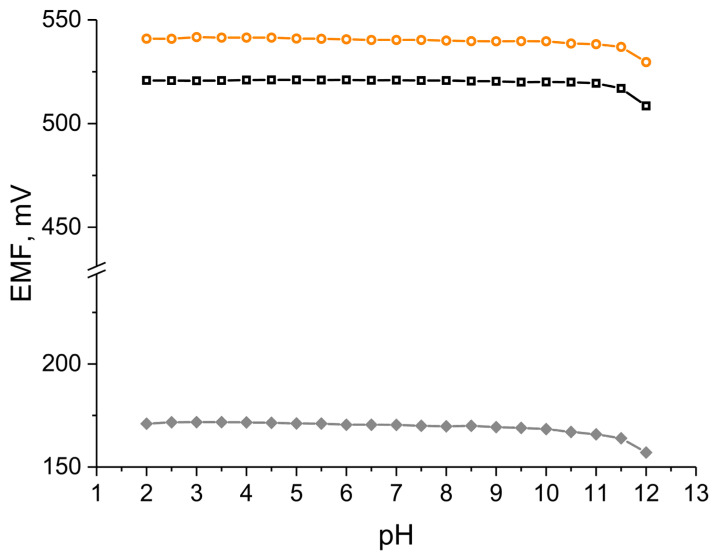
Potentiometric response recorded for GCD/K^+^-ISM (⧫), GCD/hCeO_2_ + NTs/K^+^-ISM (□) and GCD/hCeO_2_ + POT/K^+^-ISM (○) electrode during the pH test.

The pH test was conducted regardless of the presence of hydrous cerium dioxide in the composite materials, which exhibits a pH dependence [12]. The pH sensitivity test was performed for designed solid-contact electrodes in the presence of a coated disc electrode as a control group (Figure 7).

The test was carried out in solutions with constant potassium ion concentration and varying pH value (from 2.0 to 12.0). Standard 0.01 M KCl solution was titrated with sodium hydroxide (NaOH) to obtain pH-range solutions from 6–12 and hydrochloric acid (HCl) was used to obtain lower pH values. Figure 7 shows that the stable potentiometric response is achieved in the pH range between 2.0 and 11.5, which is considerably wider compared to IrO_2_ contacted electrodes [8]. According to the work by Betelu et al. [12], sensors with cerium dioxide work in wider pH range in comparison to other metal oxides of similar properties such as: TiO_2_, PtO_2_, or PdO. The test has shown that the electrodes presented in the scope of this work (based on CeO_2_) are not susceptible to changes in pH values between 2.0 and 11.5. 

## 4. Conclusions

As presented in the scope of this work, potassium-selective electrodes based on hydrous cerium dioxide as a component of solid-contact layers are an excellent example of the fact that CeO_2_ is another oxide that can be considered as an adequate material for designing this type of potentiometric sensors. 

Designed composite hCeO_2_-based materials can be characterized as superhydrophobic materials with a nanometric microstructure and high surface area. Chronopotentiometric studies showed that the addition of hydrous cerium dioxide to the carbon nanomaterial (carbon nanotubes) and the conducting polymer (poly(3-octylthiophene-2,5-diyl)) improves the electrical capacity of both materials. 

All materials were examined as solid-contact layers in potassium-selective electrodes during potentiometric tests. The best repeatability of the potentiometric response can be attributed to the GCD/hCeO_2_ + NTs/K^+^-ISM group of electrodes and the reproducibility to the GCD/hCeO_2_ + POT/K^+^-ISM group. The electrodes contacted with hCeO_2_-NTs and hCeO_2_-POT exhibit a near-Nernstian response in the K^+^ ion concentration range from 10^−6^ to 10^−1^ M.

As hCeO_2_-NTs-contacted electrodes are characterized by the highest electrical capacitance parameter, this group of electrodes exhibits the lowest drift of potentiometric response during the long-time measurement. Almost as good stability was achieved by hCeO_2_-POT-contacted electrodes thanks to the hydrophobicity of solid-contact layer material.

Although the conducting polymer (POT) tends to be susceptible to changing light conditions and hydrous cerium dioxide exhibits responsivity to pH changes, electrodes with composite materials built of these components showed insensitivity to both light exposition and changing pH values (in the pH range between 2.0 and 11.5). Designed hCeO_2_-NTs and hCeO_2_-POT-contacted electrodes are insensitive to unstable conditions that may occur during measurement, making them the reliable tools for potassium ion determination. 

The method proposed for the preparation of both composite materials and electrodes is fast and easy and does not require the use of a specific apparatus or demanding conditions. 

## Figures and Tables

**Table 1:** Calibration curves parameters calculated for one electrode representing each group after 24, 48 and 72 h of conditioning (*n* = 3).

Group of Electrodes	Resistance ± SD [kΩ]	Potential Drift ± SD [μV/s]	Capacitance ± SD [μF]
GCD/hCeO_2_/K^+^-ISM	1775 ± 37	6010 ± 60	9.4 ± 0.4
GCD/hCeO_2_ + NTs/K^+^-ISM	48.4 ± 1.9	2300 ± 50	608 ± 12
GCD/hCeO_2_ + POT/K^+^-ISM	44.3 ± 2.2	2700 ± 20	96 ± 8

**Table 2:** Potentiometric response towards K^+^ ions for studied electrodes after 24 h of electrodes’ conditioning in 0.01 M KCl (*n* = 3 items).

Group of Electrodes	Slope ± SD [mV/dec]	Standard Potential ± SD [mV]	Linear Range [M]
GCD/K^+^-ISM	58.14 ± 1.03	292 ± 5	10^−1^–10^−5^
GCD/hCeO_2_/K^+^-ISM	55.32 ± 0.52	614 ± 2	10^−1^–10^−5^
GCD/hCeO_2_ + NTs/K^+^-ISM	58.90 ± 0.13	651.8 ± 0.5	10^−1^–10^−6^
GCD/hCeO_2_ + POT/K^+^-ISM	58.21 ± 0.11	676.2 ± 0.4	10^−1^–10^−6^
